# Effect of Medetomidine and Dexmedetomidine at Different Dosages on Cat Semen Quality Using Urethral Catheterization After Pharmacological Induction (UrCaPI)

**DOI:** 10.3390/ani15040504

**Published:** 2025-02-11

**Authors:** Marco Cunto, Giulia Ballotta, Alberto Contri, Alessia Gloria, Daniele Zambelli

**Affiliations:** 1 Department of Veterinary Medical Sciences, Alma Mater Studiorum—University of Bologna, Ozzano dell’Emilia, 40064 Bologna, Italy; marco.cunto@unibo.it (M.C.); daniele.zambelli@unibo.it (D.Z.); 2 Department of Veterinary Medicine, University of Teramo, Loc. Piano d’Accio, 64100 Teramo, Italy; acontri@unite.it (A.C.); agloria@unite.it (A.G.)

**Keywords:** tomcat, semen collection, urethral catheterization after pharmacological induction, medetomidine, dexmedetomidine

## Abstract

The aim of this study was to evaluate the effectiveness of different anesthetic protocols for semen collection from healthy tomcats using Urethral Catheterization after Pharmacological Induction (UrCaPI). Twenty sexually mature tomcats (Felis catus) were included in the study, in which high and low dosages of medetomidine or dexmedetomidine were administrated to collect semen. Ten healthy mature tomcats (Group A) received high dosages of the drugs, and, in particular, five of them were i.m. injected with 120 μg/kg of medetomidine (HMED) on day 1 and with 60 μg/kg of dexmedetomidine (HDEX) after 24 h (day 2). The remaining five tomcats of this group were i.m. injected with 60 μg/kg of dexmedetomidine on day 1 and with 120 μg/kg of medetomidine on day 2. The other ten healthy mature tomcats (Group B) received a low dosage of the same drugs; the first five tomcats were i.m. injected with 50 μg/kg of medetomidine (LMED) on day 1 and with 25 μg/kg of dexmedetomidine (LDEX) on day 2; the others were i.m. injected with 25 μg/kg of dexmedetomidine on day 1 and with 50 μg/kg of medetomidine after 24 h. In both groups, semen collection was performed as soon as the pharmacological effect of the drug was reached. All protocols permitted sperm collection, even if with different results in quality for volume, concentration, total number of spermatozoa, and movement score. Results suggest that both a high dosage of medetomidine and a high dosage of dexmedetomidine could be used for the collection of good-quality semen. Semen volume μL: HMED = 32 μL (25.75–37.5), HDEX = 23 μL (15.25–28). Concentration HMED = 670 × 10^6^/mL (576–990.5), HDEX = 670.5 × 10^6^/mL (536–790). Total number of spermatozoa = 23.24 × 10^6^ (18.37–32.05). Total number of spermatozoa = 13.121 × 10^6^ (10.116–16.83). However, the protocol with dexmedetomidine could not always guarantee an adequate sedation for urethral catheterization, so using a high dosage of medetomidine is still the better way to collect high quality semen with the UrCaPI technique. The outcome of the LMED and LDEX protocols was generally unsatisfactory regarding both level of sedation and semen collection in terms of volume (LMED = 3 μL (4–7.5); LDEX = 6 μL (4–7)), concentration (LMED = 215 × 10^6^/mL (157–248); LDEX = 27.05 × 10^6^/mL (0.040–110), total number of spermatozoa (LMED = 0.88 × 10^6^ (0.581–1.38); LDEX = 0.16 × 10^6^ (0.0001–0.80), and movement score (LMED = 3 (2–3); LDEX = 2 (0–2.75)).

## Simple Summary

Twenty sexually mature tomcats were included in the study, in which high and low dosages of medetomidine or dexmedetomidine were administrated to collect semen. All protocols permitted sperm collection, even if with different results in quality for volume, concentration, total number of spermatozoa, and movement score. Results suggest that both a high dosage of medetomidine and a high dosage of dexmedetomidine could be used for the collection of good-quality semen. A high dosage of medetomidine seems to be preferable to obtain good sedation of the tomcat without association with other anesthetics and a high-quality semen with urethral catheterization after pharmacological induction. The outcome of protocols with low dosages of medetomidine and dexmedetomidine was unsatisfactory regarding both level of sedation and semen collection in terms of volume.

## 1. Introduction

Currently, semen collection and evaluation for reproductive purposes in domestic cats is a well-known procedure due to the increasing demand of feline breeding and for the use of cats as research models for wild species that are threatened by extinction [1]. The priority with any semen collection procedure is to obtain good-quality ejaculates with minimal stress for the animal [2]. Different techniques have been reported in the literature over the years: the use of an artificial vagina (AV), electroejaculation (EE), and slicing of the epididimys (EP) [3]. EE is recognized as the most reliable collection method and allows the collection of good-quality ejaculate, with the advantage that teaser queens or previous training of the tomcat is not required. On the other side, the disadvantages of EE are the requirement of specific equipment and the necessity of general anesthesia and adequate analgesia to be administered [3,4,5,6,7,8,9,10].

More recently, Urethral Catheterization after Pharmacological Induction (UrCaPI) has been reported as a simple method of semen collection [11], and is now one of most widely used semen collection techniques, both in domestic cats and wild felids [2,12,13,14].

It has been described as a non-invasive and repeatable procedure with favorable results and requires only sedation rather than anesthetization. It allows the collection of a low volume of semen with a high spermatozoa concentration, suitable for both cryopreservation and in vitro fertilization, as well as for artificial insemination [13,15,16]. Various UrCaPI protocols using medetomidine have been tested [4,13,14,15,16], and high dosages (120–130 μg/kg i.m.) are generally indicated to obtain good-quality ejaculate. A single catheterization, right after the pharmacological effect is reached, is normally enough and preferable to avoid potential trauma to the urethra, even if sometimes two or three catheterizations may be necessary in order to collect the appropriate amount of sperm for an optimal artificial insemination. Therefore, each case should be evaluated individually by the veterinarian [13]. This protocol is considered safe in healthy tomcats but, due to medetomidine action on cardiac function, a cardio-vascular examination and ECG should be performed before the procedure, and the protocol should be reserved only for cats assessed as ASA I [17].

Several studies have demonstrated that the D-isomer dexmedetomidine is the only pharmacologically active part of a 1:1 racemic mixture of medetomidine [18,19,20]. Moreover, it has been proved that dexmedetomidine, at half the dose of medetomidine, induces sedative, analgesic, cardio-respiratory, and body temperature effects comparable to those of medetomidine [21,22]. Dexmedetomidine (25 to 75 μg/kg) [23,24,25] has been successfully used to perform UrCaPI in association with other drugs, such as ketamine (5 to 10 mg/kg) [23,24] or methadone (0.2 mg/kg) [25], and alone (60 μg/kg) [26]. Using dexmedetomidine without other pharmacological agents permits one to quickly reverse the patient [26]. Furthermore, the effect of other agents on the quality of semen collected by UrCaPI have not been deeply investigated and could play an important role.

Considering all these aspects, the aim of this study is to evaluate the effects of dexmedetomidine used alone at two different dosages on sperm quality collected by UrCaPI and to compare them with two protocols previously described using medetomidine [13,27].

## 2. Materials and Methods

This study has been approved by the Scientific Ethics Committee for Experimentation on Animals of Alma Mater Studiorum—University of Bologna (Prot. N. 294336/2020), and signed informed consent was obtained from the owners of the cats before each procedure.

### 2.1. Animals

A total of twenty sexually mature tomcats (Felis catus) of different ages (mean 2.65 years ± 0.93, range 1–4 years old), weights (mean 5.48 kg ± 1.75, range 3–8 kg), and breeds (5/20 Persian, 2/20 Exotic, 3/20 British short hair, 1/20 Devon Rex, 2/20 Chartreux, 2/20 Siamese cat, 2/20 Sacred cat of Burma, 3/20 Bengal cat) were included in the study. All the toms were presented at the Reproduction Unit of Small Animal Clinical Service of the Department of Veterinary Medical Sciences—University of Bologna, between March and June, to collect semen for evaluation and/or for artificial insemination. All the animals underwent a clinical examination and were included in the study only if assessed as ASA I and with both testicles descended and palpable.

### 2.2. Experimental Design

Semen collection was performed twice, 24 h apart. The tomcats were randomly divided into two main groups of ten animals each. Group A received a high dosage of medetomidine or dexmedetomidine, and Group B received a low dosage of the same drugs, to test the effects of four pharmacological induction protocols on the quality of sperm collected by UrCaPI. In both groups, the ten cats were randomly divided into two subgroups, and the first five cats were intramuscularly injected on day 1 with medetomidine and on day 2 with dexmedetomidine, while the other five were intramuscularly injected on day 1 with dexmedetomidine and on day 2 with medetomidine. In all cases, if the tom showed an increased respiratory or cardiac frequency of more than 20% or the presence of voluntary movements, interpreted as sensitivity to the procedures, 1–4 mg of propofol (Propovet, Zoetis Italia S.r.l., Rome, Italy) was administered intravenously.

Specifically, in group A, the first five tomcats were intramuscularly injected with 120 μg/kg of medetomidine (Sedastart^®^, Ecuphar Italia S.r.l., Milano, Italy—HMED) on day 1 and with 60 μg/kg of dexmedetomidine (Sedadex^®^, Dechra Veterinary Product S.r.l., Torino, Italy)—HDEX) after 24 h (day 2). The remaining five tomcats were intramuscularly injected with 60 μg/kg of dexmedetomidine (HDEX) on day 1 and with 120 μg/kg of medetomidine (HMED) on day 2. In group B, the first five tomcats were intramuscularly injected with 50 μg/kg of medetomidine (LMED) on day 1 and with 25 μg/kg of dexmedetomidine (LDEX) after 24 h (day 2). The remaining five tomcats were intramuscularly injected with 25 μg/kg of dexmedetomidine (LDEX) on day 1 and with 50 μg/kg of medetomidine (LMED) on day 2. In all animals, medetomidine or dexmedetomidine were reversed, respectively, with half or an equal volume of atipamezole (Antisedan^®^—Zoetis) following the procedure.

### 2.3. Semen Collection and Evaluation

In all subjects, once the pharmacological effect of medetomidine or dexmedetomidine was obtained (sedation criteria: maintaining lateral recumbency even when stimulated, no response to clipper sounds, no response to clipping, no reaction or movement in response to restraint and catheterization), the semen was collected with the UrCaPI technique in preheated 0.5 mL Eppendorf tubes by introducing into the urethra a 3F urinary tomcat catheter (Portex^®^ Jackson Cat Catheter, St Paul, MN, USA), cut at the tip to remove lateral openings and to obtain a shorter open-ended catheter. As previously reported, the catheter was inserted approximately 9 cm into the urethra and immediately pulled back in order to collect by capillarity the semen released in the urethra and avoid the risk of urine reflux from the urinary bladder [13,15].

After collection, semen was evaluated for macroscopic and microscopic parameters by the same qualified operator, who did not know which anesthesiologic protocol was applied for the semen collection, to obtain the following information: volume, motility, movement score, concentration, total number of sperm cells, sperm morphology, and membrane integrity. Sperm volume was determined using a variable volume pipette, using the subtraction technique. After that, due to high concentration and low volume samples, typical of collection by UrCaPI [27], a semen sample was diluted (2 μL of sperm with 18 μL of preheated Tris-glucose-citrate) before proceeding with the remaining evaluations. According to the laboratory manual for semen assessment [28], subjective motility (0–100%) and movement score (scale of 0–5; 0 = no forward movement, 5 = steady, rapid forward progression) were estimated by the same operator, blinded for the anesthesiologic protocol or the collection technique, at 100× magnification using a phase-contrast microscope (Axiolab, Zeiss^®^, Oberkochen, Germany) equipped with a warming plate. A Bürker chamber was used for measuring sperm concentration (×10^6^/mL) and the total number of sperm cells (×10^6^/ejaculate). Finally, sperm morphology was estimated after fast green FCF-Bengal rose staining and membrane integrity after eosin-nigrosin staining using an optical microscope to count 200 sperm cells at 400× magnification [3,29].

### 2.4. Statistical Analysis

Data were analyzed using R version 3.5.2 (“Eggshell Igloo”, Copyright © 2018, The R Foundation for Statistical Computing). Descriptive statistics, including mean ± standard deviation (SD) and median and interquartile range (IQR), were calculated. All parameters were tested for normal distribution using the Shapiro–Wilk test.

Depending on their distribution, normal or not, a one-way ANOVA or Kruskal–Wallis test was used to compare semen volume (µL), concentration (×10^6^/mL), total number of sperm cells (×10^6^/vol), motility (%), forward progressive motility (0–5), normal sperm form (%), and vitality (%) between the groups. Significance was set as a *p* value of <0.05.

## 3. Results

In both groups, the UrCaPI procedure was performed about 15 min after drug administration, and all protocols permitted sperm collection in every individual. Except for total motility, normal sperm forms, and membrane integrity, sperm evaluation showed better results in group A, where high dosages of drugs were used. In addition, statistically significant differences were detected in semen quality between the groups, as described below.

The results of sperm evaluation from each group are summarized in Table 1.

### 3.1. Comparison Between High Dosages of Medetomidine (HMED) and Dexmedetomidine (HDEX)—Group A

Both pharmacological protocols used in the first experiment for the ten tomcats belonging to Group A, with 120 µg/kg of medetomidine (subgroup HMED) and 60 µg/kg dexmedetomidine (subgroup HDEX), permitted adequate sperm collection. The statistical analysis showed no significant differences between semen parameters in group A (*p* > 0.05), except for semen volume (32 μL (25.75–37.5) vs. 23 μL (15.25–28)) and total number of spermatozoa (23.24 × 10^6^/vol (18.37–32.05) vs. 13.121 × 10^6^/vol (10.116–16.83)), which turned out to be greater in subgroup HMED with respect to subgroup HDEX (*p* < 0.05). Moreover, it is important to report that in subgroup HDEX, six toms showed inadequate sedation, as slight movements of the rear limbs were noticed at the insertion of the catheter, and, as a consequence, it was necessary to administer propofol to permit urethral catheterization.

### 3.2. Comparison Between Low Dosages of Medetomidine (LMED) and Dexmedetomidine (LDEX)—Group B

Comparing all semen parameters between the two subgroups, LMED and LDEX, there were no significant differences (*p* > 0.05) except for the concentration (215 (157–248) × 10^6^/mL vs. 27.05 (0.040–110) × 10^6^/mL) and total number of spermatozoa (0.88 (0.581–1.38) × 10^6^/mL vs. 0.16 (0.0001–0.80) 10^6^/mL), which were higher in subgroup LMED (*p* < 0.05). Moreover, pharmacological administration of low dosages of both alpha-2-agonists, medetomidine, and dexmedetomidine, gave an insufficient level of sedation for urethral catheterization, which was possible only after administration of isoflurane in all cats.

### 3.3. Comparison Between High (Group A) and Low (Group B) Dosages of Medetomidine and Dexmedetomidine

Even if all pharmacological protocols permitted semen collection in all cats, data showed that there is a statistically significant difference (*p* < 0.05) in volume, concentration, total number of sperm cells, and movement score of semen collected between Group A (high dosage) and Group B (low dosage). All other parameters (total motility, sperm vitality, and normal spermatozoa) showed no significant statistical differences.

## 4. Discussion

Urethral Catheterization after Pharmacological Induction (UrCaPI) is considered a non-invasive and repeatable procedure effective for semen collection in cats [10,13,15] and wild felids [30], with favorable results. It allows collection of semen samples characterized by low volume and high concentration, suitable for both cryopreservation and in vitro fertilization, as well as for artificial insemination [13,15,16]. Different anesthesiological protocols have been proposed to perform this collection technique, with results variable and not always satisfactory [13,15,16,24,25,26]. This study aimed to evaluate the effectiveness of four anesthesiological protocols to perform UrCaPI based on high dosages of medetomidine and dexmedetomidine (120 and 60 µg/kg, respectively—group A) and low dosages of the same drugs (50 and 25 µg/kg, respectively—group B).

Both experiments demonstrated that sperm collection from healthy tomcats is possible using either medetomidine or dexmedetomidine, at different dosages, using the UrCaPI technique, with semen characteristics, in particular for group A, comparable to the values reported in the literature [10,13,15,16,25,26], despite differences in the techniques used for semen motility assessment. In the present study, subjective motility was assessed by an experienced operator. Although CASA systems allow the detection of subtle differences in sperm kinetics [31], the subjective estimation of sperm motility percentage is widely diffused due to the significant agreement between subjective and objective motility in feline semen previously demonstrated [32]. This procedure, as previously reported [10,13,15,16,25,26], permitted one to collect high concentration and low volume samples, as the use of an alpha-2-adrenergic agonist seems to induce ejaculation with reduced contribution to the ejaculate from the accessory glands. For that reason, it may be necessary to dilute the sample before evaluating for macroscopic and microscopic parameters. To obtain a high-quality semen, it is important to correctly catheterize the tomcat, as an incorrect procedure could induce urinary reflux within the catheter. For that reason, the catheter was inserted in urethra for about 9 cm after pulling back the prepuce during the procedure; this way permits one to reach the prostatic urethra, avoiding urine reflux. Specifically, in this study, both HMED (120 μg/kg) and HDEX (60 μg/kg) protocols permitted the collection of good-quality semen, proving that dexmedetomidine is a possible alternative to medetomidine, which was the sedative of choice in felids to perform UrCaPI, but which is no longer available in many countries. However, it is necessary to take into consideration that semen volume and total sperm count were higher in the medetomidine subgroup (HMED) compared to dexmedetomidine subgroup (HDEX). In a study by Burton et al. [26], total sperm count in samples collected using the same dosage of dexmedetomidine alone (60 µg/kg) was slightly higher (17.91 × 10^6^) than total sperm count in the HDEX group, but still lower than total sperm count in the HMED group, confirming best results using medetomidine. Nevertheless, it cannot be excluded that these differences are due to the different cat breeds involved in the two studies.

Moreover, while the HMED protocol always guaranteed an adequate sedation, in some cases (n = 6), the HDEX protocol did not permit performing the catheterization procedure due to slight movements of the rear limbs, which made it necessary to administer propofol to proceed with the semen collection by UrCaPI. This issue has not been reported by Burton et al. [26].

Our results are not in line with those reported in a paper by Madrigal-Valverde et al. [24], where, using high dosages of medetomidine and low dosages of dexmedetomidine, the quality of sperm samples collected by UrCaPI and adequacy of sedation was good and comparable between the two protocols. The better results obtained by Madrigal-Valverde et al. [24] regarding adequacy of sedation may be justified by the use of ketamine in association with dexmedetomidine, but the absence of differences in sperm quality between the two protocols different from what was observed in our study is interesting and could be further investigated. It is reasonable not to attribute the better sperm quality obtained by Madrigal-Valverde et al. [24] to the association with ketamine in their protocol, which, used alone, has been previously related to a minor number of spermatozoa being displaced in the urethra [27].

The outcomes of the LMED (50 μg/kg) and LDEX (25 μg/kg) protocols were better for LMED considering concentration and total number of spermatozoa, but results were generally unsatisfactory regarding both semen collection and level of sedation. In fact, using these protocols, a low quality of the sperm in terms of volume, concentration, total number of spermatozoa, and quality of the movement was observed. Therefore, based on our results, sperm collected with lower doses of medetomidine will most likely be less efficient in procedures such as in vivo fertilization and cryopreservation, therefore limiting its use to in vitro procedures only. Results obtained using the LMED protocol are in line with data previously published [13], confirming, at least using medetomidine, the necessity to administer high dosages of this drug to collect higher quality semen in cats. As hypothesized considering dexmedetomidine’s chemical structure and mechanism of action, the LDEX protocol permitted the collection of low quality semen, with results similar to those obtained with the LMED protocol, even if worse than those reported by Madrigal-Valverde et al. [24]. 

## 5. Conclusions

In conclusion, currently, a high dosage of medetomidine seems to be the preferable choice to obtain good sedation of tomcats without association with other anesthetics and a high-quality semen with urethral catheterization after pharmacological induction. Using the HDEX protocol, high-quality semen collection is possible, but, in our experience, the use of anesthetics is suggested to obtain a good grade of sedation. Even if low dosages of dexmedetomidine have been reported to permit the collection of good-quality sperm using UrCaPI [24], our data are different, showing unsatisfactory results regarding both sperm quality and sedation adequacy using LMED and LDEX protocols. However, further studies with low dosages of dexmedetomidine could be useful to understand the differences reported in quality of sperm collected using this protocol.

## Figures and Tables

**Table 1:** Descriptive statistics for evaluated fresh sperm parameters (*n* = 20) collected after UrCaPI with 120 µg/kg of medetomidine (A-HMED), 60 µg/kg of dexmedetomidine (A-HDEX), 50 µg/kg of medetomidine (B-LMED), and 25 µg/kg of dexmedetomidine (B-LDEX). Different letters between groups indicate significant difference (*p* value < 0.05). Parametric and non-parametric data are ex-pressed, respectively, as mean ± standard deviation or median (IQR).

	A-HMED	A-HDEX	B-LMED	B-LDEX
Semen volume (μL)	32 (25.75–37.5) ^A^	23 (15.25–28) ^A-B^	3 (4–7.5) ^A-B^	6 (4–7) ^A-B^
Concentration (×10^6^/mL)	670 (576–990.5) ^A^	670.5 (536–790) ^B^	215 (157–248) ^A-B-C^	27.05 (0.04–110) ^A-B-C^
Total n° Spt (×10^6^/vol)	23.24 (18.37–32.05) ^A^	13.12 (10.12–16.83) ^A-B^	0.88 (0.58–1.38) ^A-B-C^	0.16 (0.0001–0.80)^A-B-C^
Motility (%)	50 (42.5–60)	50 (39.75–58.75)	50 (40–57.5)	40 (0–50)
Movement score (0–5)	4 (4–4.75) ^A^	4 (3.25–4) ^B^	3 (2–3) ^A-B^	2 (0–2.75) ^A-B^
Normal sperm forms (%)	51.32 ± 17.34	48.42 ± 24.19	52.9 ± 11.44	37.8 ± 26.98
Vitality (%)	65 (56.25–67.5)	60 (56.25–65.75)	69 (65.25–71.50)	61 (0–65.5)

## Data Availability

The data presented in this study are available on request from the corresponding author.
